# Supplementary material for: Distinct UPR and Autophagic Functions Define Cell-Specific Responses to Proteotoxic Stress in Microglial and Neuronal Cell Lines
Source: Cells. 2024 Dec 15;13(24):2069. doi: 10.3390/cells13242069 (PMC11674117; doi:10.3390/cells13242069)
Supplement: Supplementary file 1 [file cells-13-02069-s001.zip › Data Sheet 1.PDF]

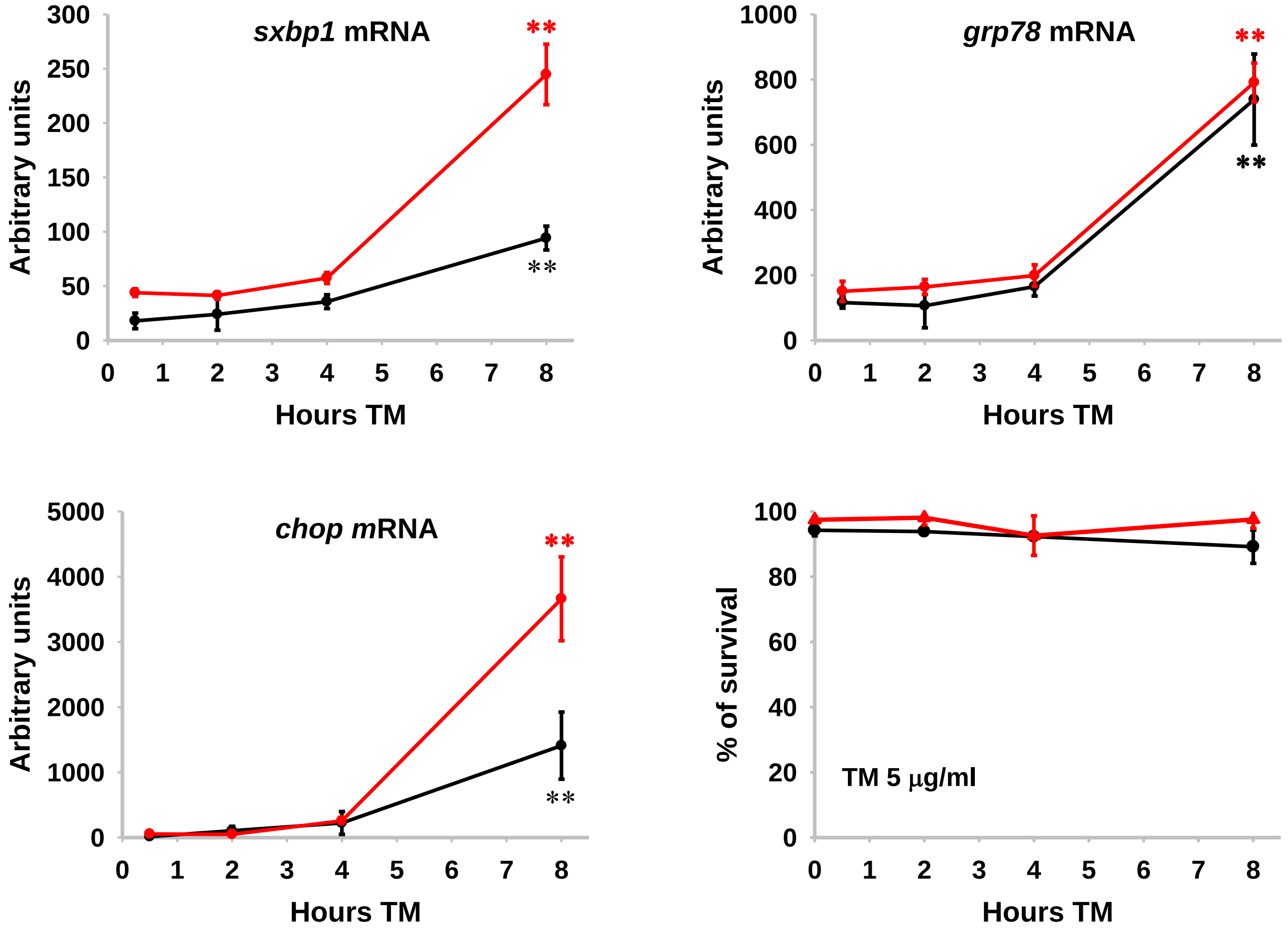

Supplementary Figure 1. Induction of ER stress by tunicamycin for 2, 4 and 8 hours. The analysis of the transcriptional induction of the UPR down-stream factors by qPCR showed that tunicamycin treatments led to complete UPR activation (*sxbp1*, *grp78* and *chop* mRNA expression) in both cell lines. Data are expressed as arbitrary units of fold change for the gene expression or as mean of the percentage of the cell survival. Statistical significance **p<0.01.No differences were observed in cell survival. Experiments were repeated at least 4 times.
