# Supplementary material for: Distinct UPR and Autophagic Functions Define Cell-Specific Responses to Proteotoxic Stress in Microglial and Neuronal Cell Lines
Source: Cells. 2024 Dec 15;13(24):2069. doi: 10.3390/cells13242069 (PMC11674117; doi:10.3390/cells13242069)
Supplement: Supplementary file 1 [file cells-13-02069-s001.zip › Data Sheet 2.PDF]

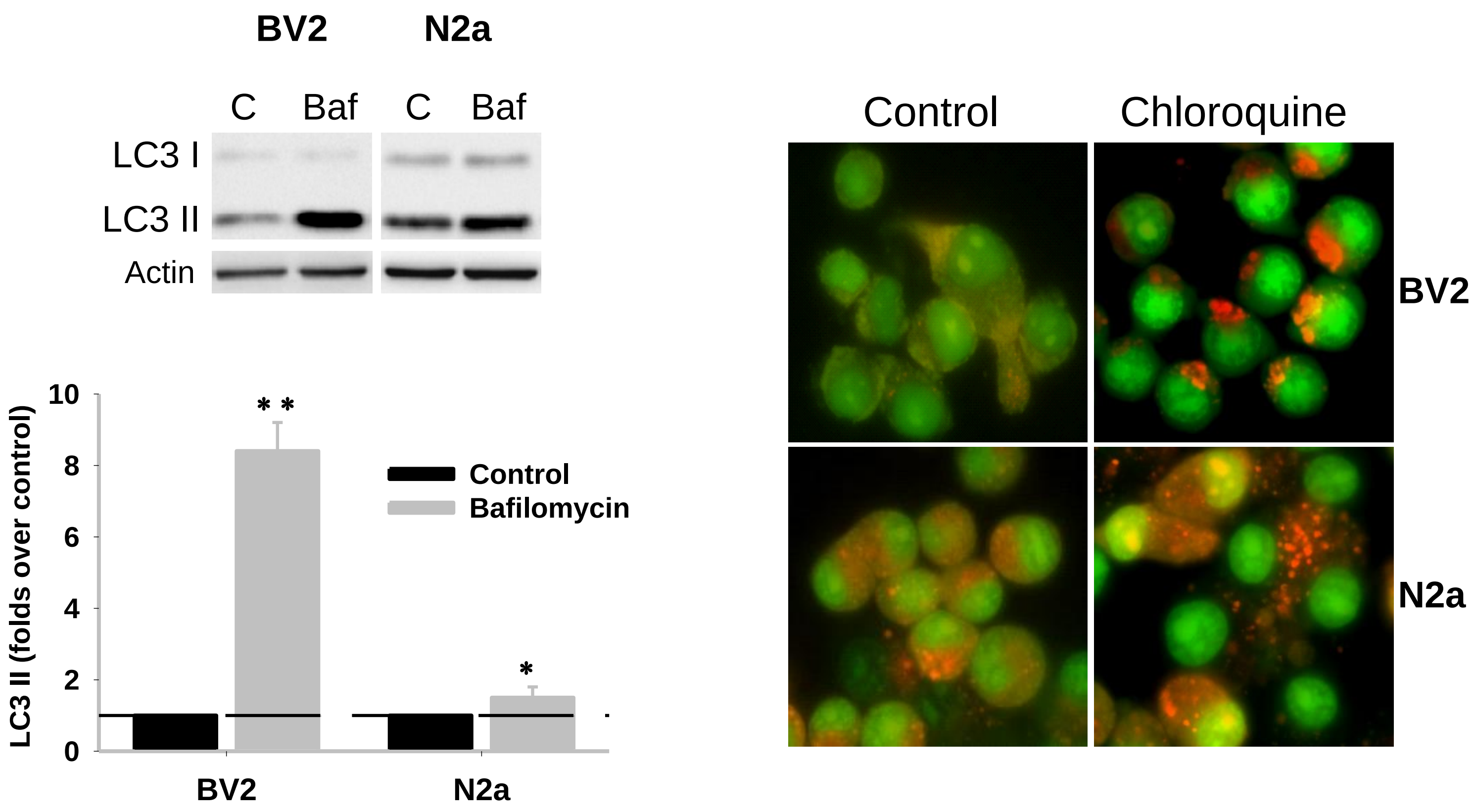

Supplementary Figure 2. Analysis of autophagy flux by bafilomycin and chloroquine treatments. It is shown the representative image of LC3II western-blot of both cell lines, with and without bafilomycin for 6 hours. It is also provided the quantification of LC3II, suggesting a higher and/or faster basal autophagic activity in BV2 compared to N2a. This is supported by the acridine orange staining (which mostly labels lysosomes compartments). Data are expressed as fold of control of OD ± SD. Statistical significance *p<0.05 and **p<0.01. Experiments were repeated 3 times.
