# Supplementary material for: Distinct UPR and Autophagic Functions Define Cell-Specific Responses to Proteotoxic Stress in Microglial and Neuronal Cell Lines
Source: Cells. 2024 Dec 15;13(24):2069. doi: 10.3390/cells13242069 (PMC11674117; doi:10.3390/cells13242069)
Supplement: Supplementary file 1 [file cells-13-02069-s001.zip › Data Sheet 3.PDF]

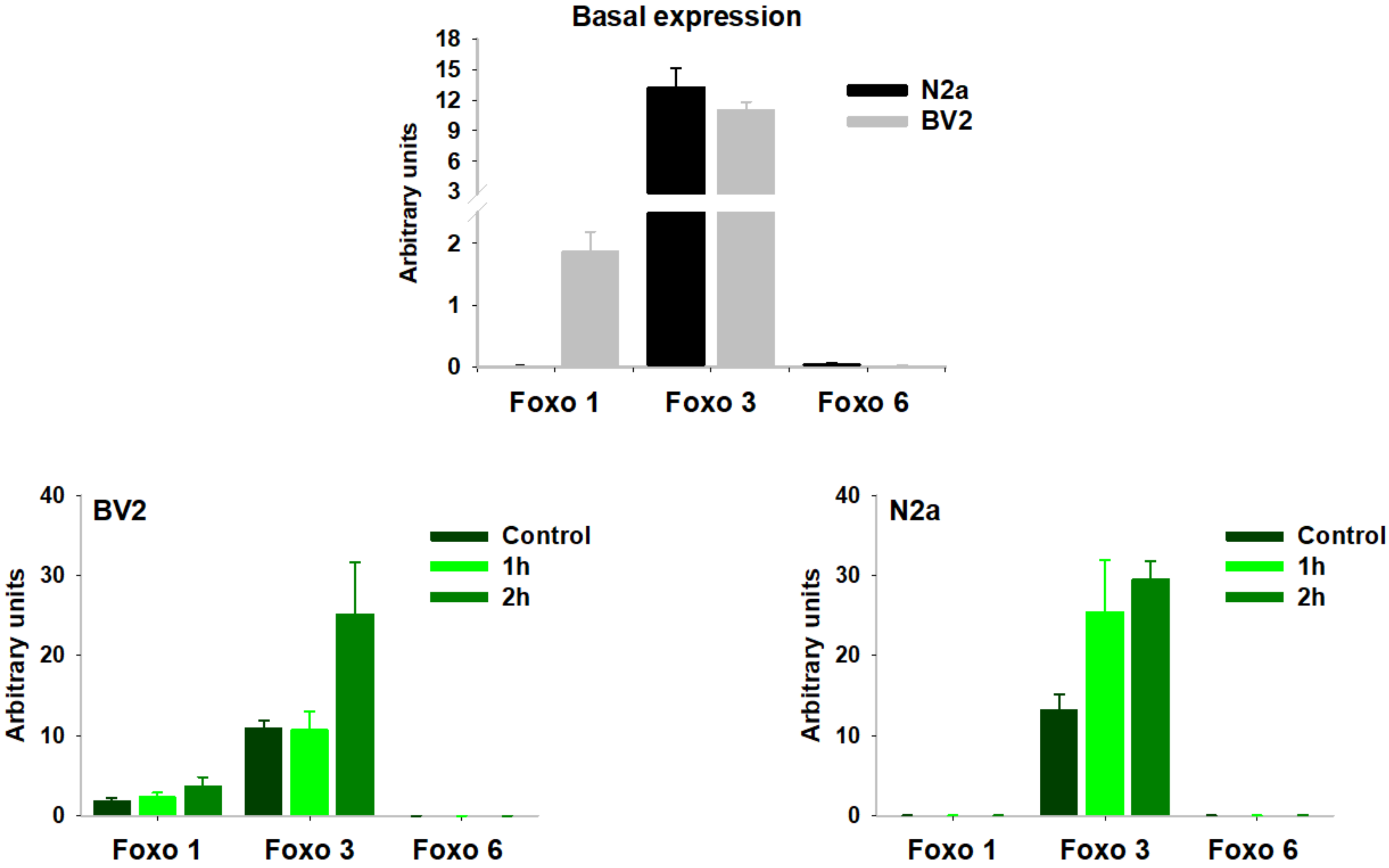

Supplementary Figure 3. Analysis of the transcriptional expression of *foxo1*, *foxo3* and *foxo6*. The basal expression of these genes showed a differential expression among these cells, with a predominance of *foxo3* in N2a, and *foxo1* and *foxo3* in BV2 cells. There was also a differential transcriptional regulation of *foxo* genes by proteotoxic stress among BV2 and N2a cells. Data are expressed as arbitrary units of fold change ± SD for each condition. Experiments were repeated at least 4 times.
