# Supplementary material for: Distinct UPR and Autophagic Functions Define Cell-Specific Responses to Proteotoxic Stress in Microglial and Neuronal Cell Lines
Source: Cells. 2024 Dec 15;13(24):2069. doi: 10.3390/cells13242069 (PMC11674117; doi:10.3390/cells13242069)
Supplement: Supplementary file 1 [file cells-13-02069-s001.zip › Data Sheet 4.PDF]

Supplementary figure 4

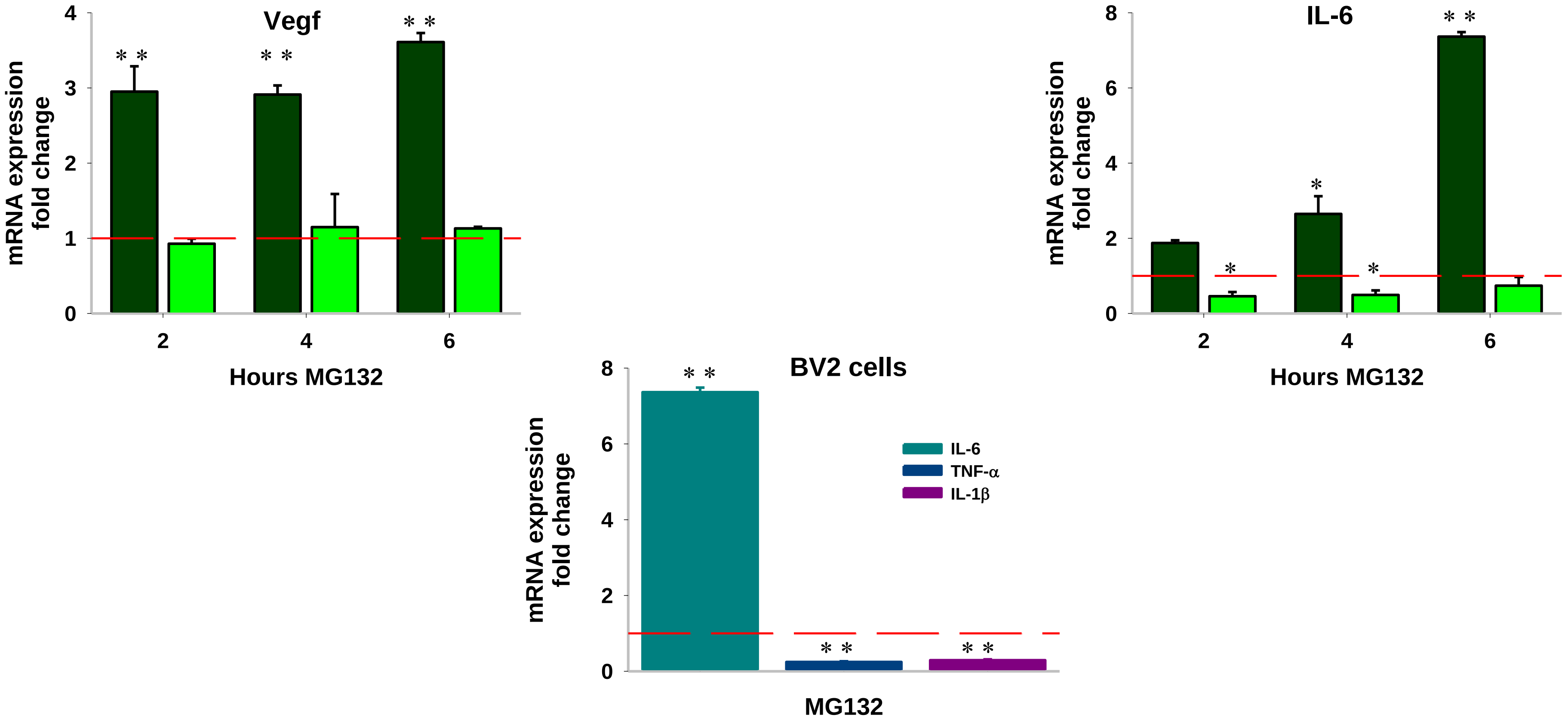

Supplementary Figure 4. Analysis by qPCR of the expression of genes regulated by the Wnt/β-catenin pathway and other pro-inflammatory cytokines following MG132 treatment. mRNA expression of *Vegf* and *IL-6* genes following proteasome inhibition. mRNA expression of *IL-6* together with the pro-inflammatory cytokines *TNF-α* and *IL-1β* in BV2 cells. Proteasome inhibition induced a significant transcriptional upregulation of *Vegf* and *IL-6* genes exclusively in BV2 cells. Data are expressed as arbitrary units of fold change related to control ± SD. Statistical significance *p<0.05 and **p<0.01. Experiments were repeated at least 4 times.
