# Supplementary material for: Distinct UPR and Autophagic Functions Define Cell-Specific Responses to Proteotoxic Stress in Microglial and Neuronal Cell Lines
Source: Cells. 2024 Dec 15;13(24):2069. doi: 10.3390/cells13242069 (PMC11674117; doi:10.3390/cells13242069)
Supplement: Supplementary file 1 [file cells-13-02069-s001.zip › Data Sheet 5.PDF]

Supplementary figure 5

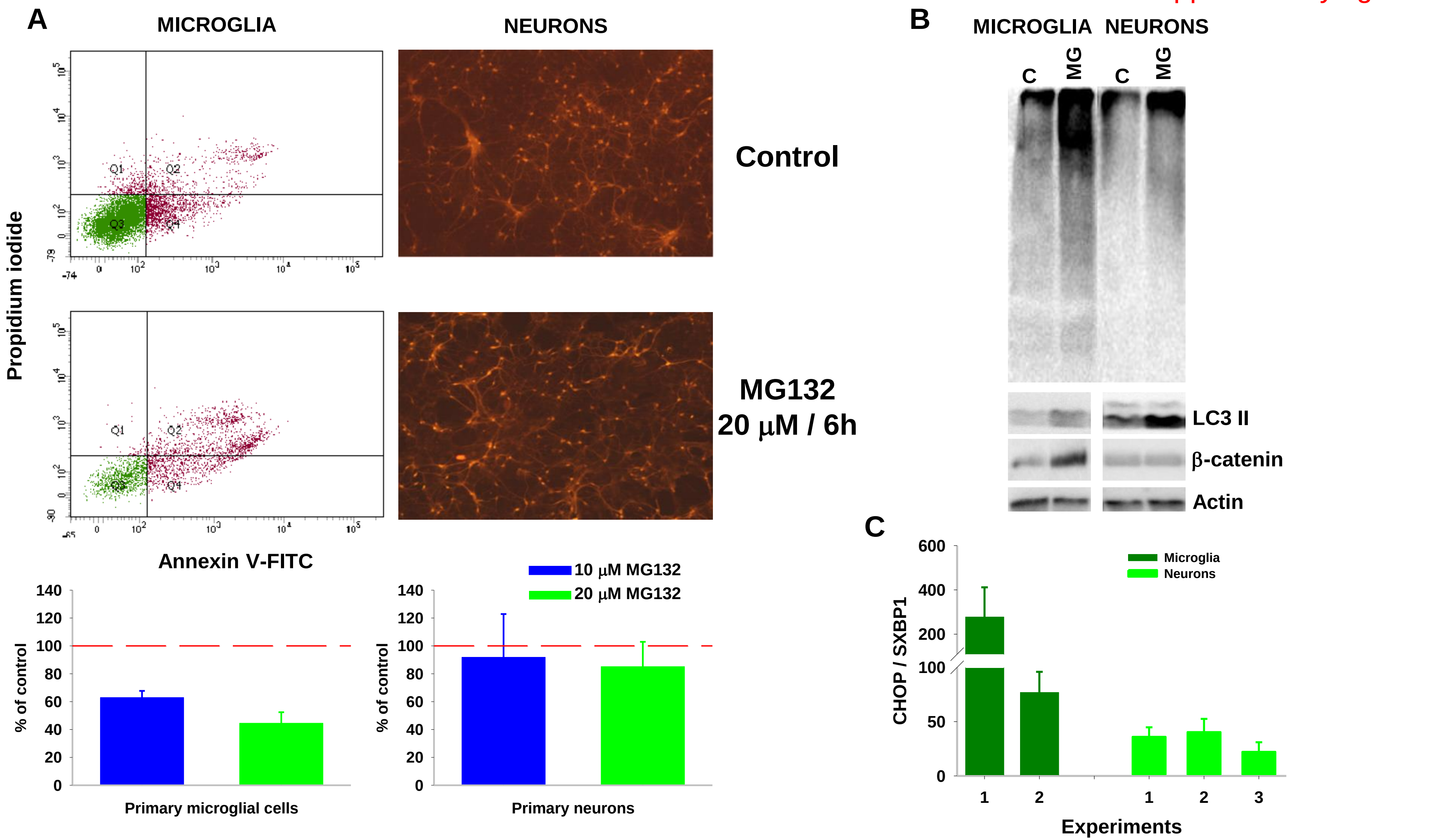

Supplementary Figure 5. Differential effect of proteotoxic stress in primary microglial and neuronal cell cultures. **A.** Cell viability was assessed in both primary microglial (n=2), and primary neuronal cell cultures (n=3), incubated with 10 and 20 μM of MG132 for 6 hours. Microglial cells were more sensitivity to proteotoxic stress than neurons. In particular, microglial cells reduced cell viability in a dose-dependent manner (around 40% and 55% for 10 and 20 μM of MG132, respectively). Doses of 1 and 5 μM of MG132 for 8 hours did not affect either neuronal or microglial cell viability after 6 hours of incubation. **B**. Proteotoxic stress (20 μM MG132 for 6 hours), produced higher accumulation of high-molecular-weight poly-ubiquitinated proteins in microglial cells. On the contrary, LC3-II accumulation was higher in neurons than in microglial cells. Microglial cells accumulated β-catenin, but not neurons. C. Shown is the mRNA *chop/sxbp1* ratio of individual experiments (n=2 microglial cells; and n=3 neuronal cells). Microglial cells showed a higher chop/sxbp1 ratio than neurons, in 2 out of 2 experiments, supporting a predominant activation of the PERK-CHOP over the IRE1a-sXbp1pathway in microglial cells, and the contrary in neurons. Statistical analysis was not performed due to the low number of experiments for microglial cells.

Primary cell cultures protocol

Primary microglia cultures were prepared from CD1 mice pups at postnatal day P1-P2 as previously described by Jimenez et al., 2008 (Jimenez et al., 2008. J Neurosci. 28(45):11650-61. doi: 10.1523/JNEUROSCI.3024-08.2008). Briefly, brains were enzymatically and mechanically dissociated in trypsin-EDTA 1x (Biowest). We used a 40 μm filter to eliminate debris and seed the cells at a 250,000 cells/ml density. Mixed astrocytic/microglial cells were cultured in DMEM-F12, supplemented with 2 mM glutamine, 10% (v/v) fetal bovine serum, non-essential amino acids (1×), plus gentamycin (10 μg/ml), penicillin (100 U/ml) and streptomycin (100 μg/ml). After ~ 18 days, we incubated with 0.25% trypsin, 1mM EDTA, DMEM-F12 and detached astrocytes were removed. The next day, enriched microglia culture were incubated for 6 hours with different doses of MG132 (10 and 20 μM). Cell viability was assessed using an Apoptosis detection kit (Inmunostep) and flow cytometry (n=2).

Primary **neuronal cultures** were obtained from the hippocampus of E17 embryonic CD1 mice, as previously described by Hinojosa et al., 2022 (Hinojosa MG., et al., 2022 26;14(3):175. doi: 10.3390/toxins14030175. PMID: 35324672; PMCID: PMC8950865). Dissected hippocampus were dissociated in 41 U/ml papain and 50,000 cells/well were seeded in 12 mm diameter coverslips previously coated with poly-D-lysine in 24-well plates. Cells were maintained in neuronal medium containing neurobasal, B27 (2%), Glutamax (2 mM), and penicillin and streptomycin (50 mg/mL). After 18 days, cells were incubated for 6 hours with different doses of MG132 (10 and 20 μM), and cell viability was assessed by counting of double-positive cells for MAP2 and NeuN antibodies (n=3). Briefly, the cells were fixed with paraformaldehyde (4%) for 20 min at room temperature, washed twice with PBS, and permeabilized by incubation with 0.1% Triton-X 100 in PBS for 1 h at room temperature. Then, cells were incubated with blocking solution (0.1% Triton-X 100 and 1% bovine serum albumin (BSA) in PBS) for 3 h, following by the primary antibodies rabbit anti-MAP2 (AB5622, Sigma-Aldrich, 1:1,000) and mouse anti-NeuN (clone A60, Sigma-Aldrich, 1:1,000) overnight incubation at 4 ºC. Finally, samples were washed with 0.1% Triton-X 100 in PBS and incubated for 1 hour with the secondary antibodies (1:500 dilution of Alexa Fluor™ 546 donkey anti-rabbit IgG and 488 donkey anti-mouse IgG). Finally, samples were washed twice with PBS, and the coverslips were added to the slides with Fluorescent Mounting Medium. Images were obtained by Zeiss apotome epifluorescence microscope at 20X to capture viability images and analyzed using ImageJ (FIJI) and the plugins “Cell Counter” to quantify the number of neurons.
